# Hierridin B Isolated from a Marine Cyanobacterium Alters VDAC1, Mitochondrial Activity, and Cell Cycle Genes on HT-29 Colon Adenocarcinoma Cells

**DOI:** 10.3390/md14090158

**Published:** 2016-08-31

**Authors:** Sara Freitas, Rosário Martins, Margarida Costa, Pedro N. Leão, Rui Vitorino, Vitor Vasconcelos, Ralph Urbatzka

**Affiliations:** 1 Interdisciplinary Center of Marine and Environmental Research (CIIMAR/CIMAR), University of Porto, Rua dos Bragas 289, 4050-123 Porto, Portugal; freitas.srf.09@gmail.com (S.F.); mrm@estsp.ipp.pt (R.M.); costa.anamarg@gmail.com (M.C.); pleao@ciimar.up.pt (P.N.L.); vmvascon@fc.up.pt (V.V.); 2 Health and Environmental Research Center (CISA), School of Apllied Health Sciences of Porto, Polytechnic Porto, Rua Valente Perfeito 322, 4400-330 Vila Nova de Gaia, Portugal; 3 Department of Biology, Faculty of Sciences, University of Porto, Rua do Campo Alegre, Edifício FC4, 4169-007 Porto, Portugal; 4 Institute for Molecular and Cell Biology (IBMC), University of Porto, Rua do Campo Alegre 823, 4150-180 Porto, Portugal; 5 Department of Medical Sciences, Institute of Biomedicine—iBiMED, University of Aveiro, 3810-193 Aveiro, Portugal; rvitorino@ua.pt; 6 Department of Physiology and Cardiothoracic Surgery, Faculty of Medicine, University of Porto, 4200-319 Porto, Portugal

**Keywords:** natural products, bioactive compounds, anticancer drugs, colon cancer cells, marine cyanobacteria, mitochondria, vdac1, cell cycle, proteomics, high content analysis

## Abstract

Background: Hierridin B was isolated from a marine cyanobacterium *Cyanobium* sp. strain and induced cytotoxicity selectively in HT-29 adenocarcinoma cells. The underlying molecular mechanism was not yet elucidated. Methods: HT-29 cells were exposed to the IC_50_ concentration of hierridin B (100.2 μM) for 48 h. Non-targeted proteomics was performed using 2D gel electrophoresis and MALDI-TOF/TOF mass spectrometry. The mRNA expression of apoptotic and cell cycle genes were analyzed by real-time PCR. Automated quantification of 160 cytoplasm and mitochondrial parameter was done by fluorescence microscopy using CellProfiler software. Results: Proteomics identified 21 significant different proteins, which belonged to protein folding/synthesis and cell structure amongst others. Increase of VDAC1 protein responsible for formation of mitochondrial channels was confirmed by mRNA expression. A 10-fold decrease of cytoskeleton proteins (STMN1, TBCA) provided a link to alterations of the cell cycle. CCNB1 and CCNE mRNA were decreased two-fold, and P21CIP increased 10-fold, indicative of cell cycle arrest. Morphological analysis of mitochondrial parameter confirmed a reduced mitochondrial activity. Conclusion: Hierridin B is a potential anticancer compound that targets mitochondrial activity and function.

## 1. Introduction

Cancer is one of the leading causes of death in the world and the ability of resistance adaptation towards many drug treatments and less toxic side effects requires the further development of novel drugs [1]. Colon cancer causes high mortality worldwide arising from genetic mutations in gastrointestinal epithelial cells, altering its normal functioning and proliferation [2,3]. The incidence of colorectal cancer is directly related with diet plans and overweight problems among populations, due to the excessive presence of meat and alcohol in routine meals and lack of fruits and vegetables [4].

The marine environment is a prolific source of secondary active metabolites, which have been isolated from organisms as sponges, tunicates, algae, and cyanobacteria [5,6]. Cyanobacteria are an ancient group of photoautotrophic organisms and are known to produce anticancer, anti-inflammatory, and antibacterial agents, among others [7,8,9,10]. Picocyanobacteria have been described in recent years as a promising resource for the isolation and purification of compounds with pharmacological activity, mainly as anti-cancer agents [11,12,13]. One example is hierridin B, a compound produced by the picoplanktonic cyanobacterial strain *Cyanobium* sp. LEGE 06113, isolated from the Portuguese coast by a bioassay-guided fractionation approach [14]. Hierridin B demonstrated a growth inhibitor/cytotoxic effect selectively on the adenocarcinoma cell line HT-29 with an IC_50_ value of 100.2 μM; no cytotoxic effects were reported for other cancer cell lines as HEPG2, MG63, RKO, SHSY5Y, SKBR3, T47D, or for normal prostate epithelium cells PNT2 [14]. Compounds isolated from efficient phenotypic screening assays require searching for possible biological targets to characterize the underlying mechanisms and altered pathways [15].

Consequently, the aim of the present study was to advance the knowledge regarding the growth inhibitory/cytotoxic effect of hierridin B on the colon adenocarcinoma cell line HT-29. Non-targeted proteomics was performed to gain insights into altered proteins, and the mRNA expression of cell cycle and apoptosis genes were quantified. Since results pointed to an involvement of mitochondrial proteins in the observed cytotoxicity, fluorescent microscopy analysis was performed with a CellProfiler-based quantification of morphological alterations to the cytoplasm and mitochondria.

## 2. Results

### 2.1. Protein Expression

**Figure 1:**
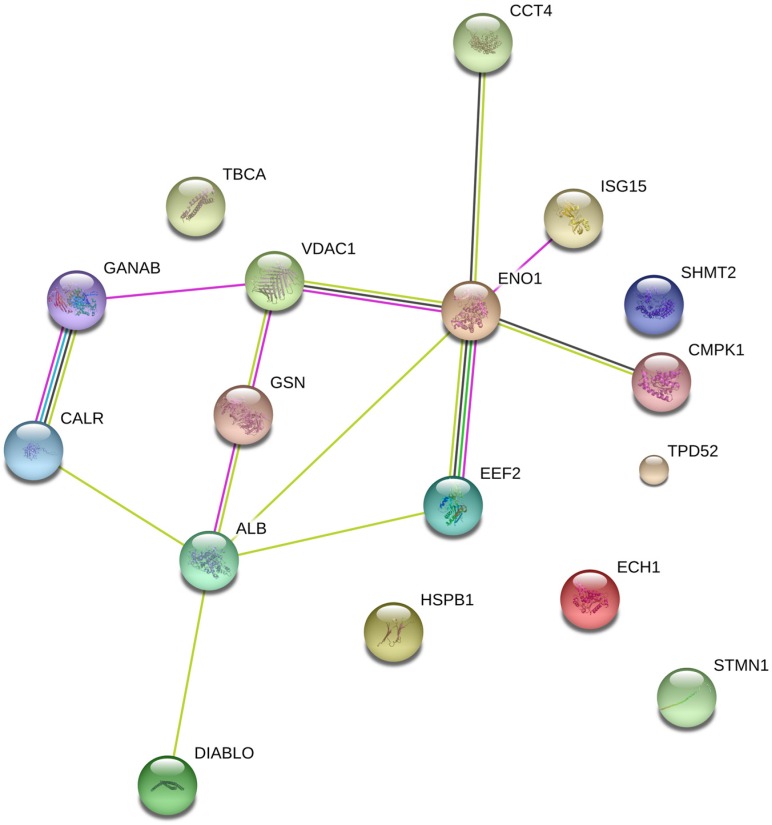
Protein interaction network for significant different proteins after exposure to hierridin B in HT-29 colon carcinoma cells.

To analyze the selective cytotoxic mechanisms of hierridin B in the HT-29 cell line, a non-targeted proteomic analysis was performed using two-dimensional gel electrophoresis (2DGE). The analysis of 2DGE gels by the software PDQuest (BioRad, Hercules, CA, USA) revealed differences between the solvent control group (dimethylsulfoxide, DMSO) and exposure to hierridin B. Twenty-one significant spots were positively identified by matrix assisted laser desorption/ionization-time of flight/time of flight (MALDI-TOF/TOF) mass spectrometry (Table 1), while four different spots could not be identified. Network analyses (Figure 1) demonstrated the connection between proteins involved in protein folding/protein synthesis (neutral alpha-glucosidase AB, GANAB; calreticulin, CALR; t-complex protein 1 subunit delta, TCPD; elongation factor 2, EEF2) to mitochondrial (voltage-dependent anion-selective channel protein 1, VDAC1) and cell structure (gelsolin, GSN; t-complex protein 1 subunit delta, TCPD) proteins, which were linked to glycolysis (alpha-enolase, ENO1) and pyrimidine biosynthesis (UMP-CMP kinase, CMPK1). Outside of the predicted network based on known interaction of proteins, further cell structural proteins were present (tubulin-specific chaperone A, TBCA; heat-shock protein beta-1, HSPB1; stathmin, STMN1), as well as proteins for tumor survival (serine hydroxymethyl transferase, SHMT2), cell proliferation (tumor protein D52, TPD52), or fatty acid metabolism (delta(3,5)-delta(2,4)-dienoyl-CoA isomerase, ECH1).

**Figure 2:**
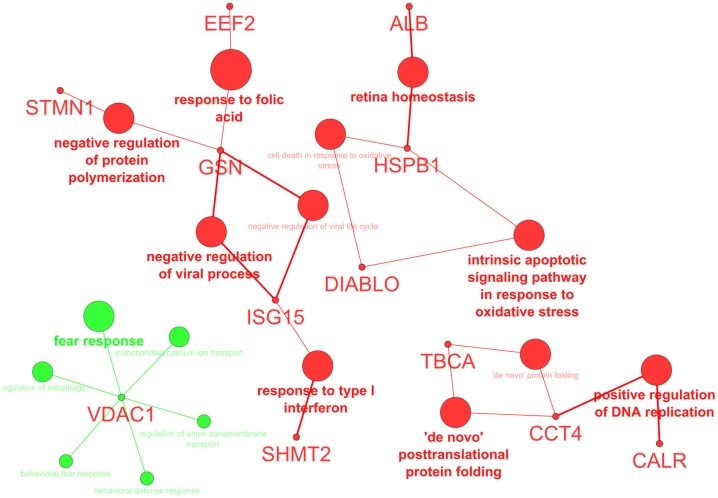
Biological processes (BP) altered by hierridin B treatment; green color indicates an increase, while red color a decrease of BP.

Analysis of the biological processes confirmed the prevalence of “mitochondrial calcium ion transport” and “regulation of mitophagy”, supported by VDAC1, whereas “‘de novo’ posttranslational protein folding”, “positive regulation of DNA replication”, and “intrinsic apoptotic signaling pathway in response to oxidative stress” were decreased by hierridin B treatment (Figure 2). 

### 2.2. mRNA Expression of Target Genes

**Figure 3:**
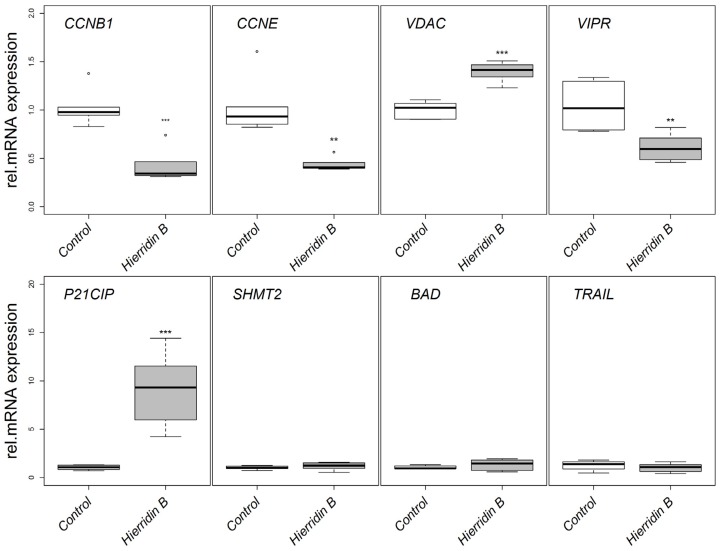
Relative mRNA expression in the HT-29 adenocarcinoma cell line in response to hierridin B exposure. Data are presented as box-whisker plots (box: 25%–75% percentile, outer bars: 100% percentile without outliers, inner bar: median, cross: mean). The mRNA expression was normalized to the geometrical mean of ribosomal protein L8 (RPL8) and hypoxanthine phosphoribosyl transferase (HPRT1). Statistical differences are indicated by asterisks; * = *p* < 0.05, ** = *p* < 0.01, *** = *p* < 0.001.

The mRNA expression of target genes involved in apoptosis (BCL2-associated agonist of cell death, BAD; tumor necrosis factor superfamily, member 10, TRAIL), cell cycle (cyclin B1, CCNB1; cyclin E1, CCNE; cyclin-dependent kinase inhibitor 1A, P21CIP) and mitochondria regulation (voltage-dependent anion channel 1, VDAC), tumor survival (serine hydroxymethyl transferase 2, SHMT2), and proliferation (vasoactive intestinal peptide receptor 1, VIPR) were analyzed by real-time PCR using a multiple reference gene normalization approach [16]. CCNB1 and CCNE decreased its mRNA expression two-fold in response to hierridin B, while P21CIP increased 10-fold (Figure 3). The mRNA expression of VDAC increased 1.5-fold, while VIPR decreased two-fold (Figure 3). BAD, TRAIL, and SHMT2 mRNA levels did not show any significant alterations.

### 2.3. Fluorescence Microscopy Analysis

**Figure 4:**
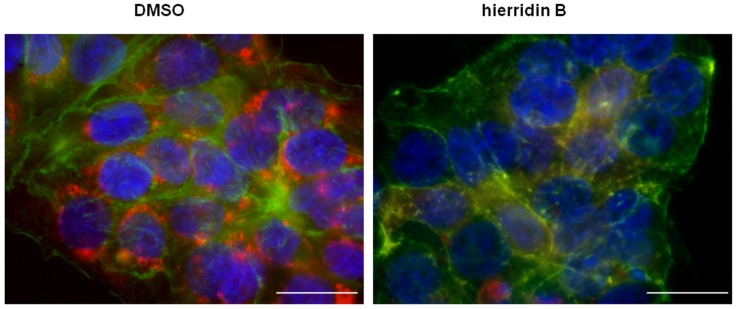
Overlay of three fluorescent channels (blue, nucleus, Hoechst 33342; green, cytoplasm, acti-stain 488; red, mitochondria, MitoTracker CMXROS) from HT-29 colon adenocarcinoma cells exposed to solvent control (DMSO) and hierridin B. Scale bar corresponds to 20 μm.

**Figure 5:**
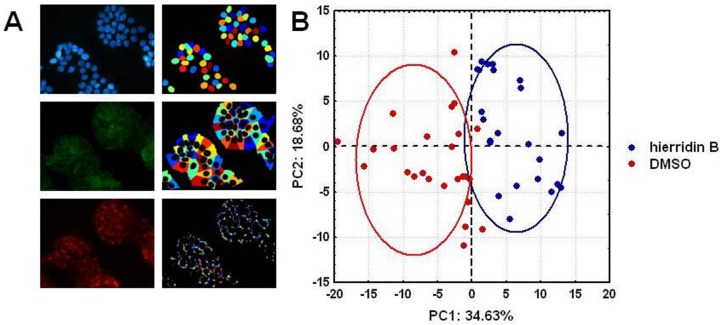

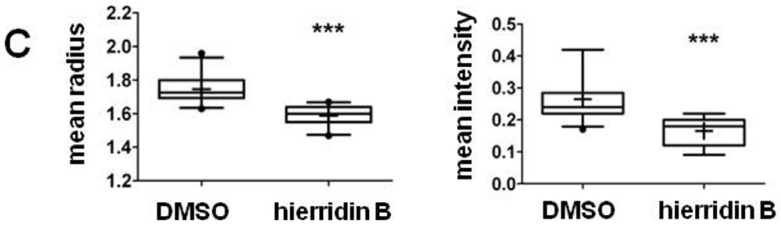
(**A**) Fluorescent images (blue, nucleus; green, cytoplasm; red, mitochondria) and automated analysis by CellProfiler software; (**B**) principal component analysis of 160 parameters (object intensity, object radial distribution, size and shape of objects, and texture) from cytoplasm and mitochondria quantified by CellProfiler. Principal component 1 (PC1) discriminated between DMSO (solvent control, 1%) and hierridin B treatment. Factors that mainly contributed to the (PC1) were mitochondrial parameters, while cytoplasm parameters mainly contributed to PC2; and (**C**) detailed analysis of two mitochondrial parameters, area shape mean radius and fluorescence mean intensity. Data are represented as box-whisker plots, and statistical differences are indicated by asterisks (*** = *p* < 0.001, Mann-Whitney U-test).

The fluorescence microscopy analyses demonstrated a strong effect of hierridin B on mitochondria in the HT-29 cells, stained with Hoechst 3342 (nucleus), F-actin 488 (cytoplasm), and MitoTracker CMX ROS (mitochondria, Figure 4). The exposure to hierridin B resulted in a substantial lower staining of mitochondria. An automated quantification of cytoplasm and mitochondria was performed using CellProfiler software and, in total, 160 parameters were measured. Figure 5A demonstrates the recognition of cellular structures by CellProfiler. Principal component analysis discriminated between DMSO (solvent control, 1%) and hierridin B treatment (100.2 μM), majorly by principal component 1 (PC1), Figure 5B. Factors that contributed mainly (>0.85) to PC1 were mitochondrial-related parameters (AreaShape_MaximumRadius, AreaShape_MeanRadius, AreaShape_MedianRadius, AreaShape_MinorAxisLength, Intensity_IntegratedIntensityEdge, Intensity_IntegratedIntensity, Intensity_LowerQuartileIntensity, Intensity_MADIntensity, Intensity_MassDisplacement, Intensity_MaxIntensityEdge, Intensity_MaxIntensity, Intensity_MeanIntensityEdge, Intensity_MeanIntensity, Intensity_MedianIntensity, Intensity_MinIntensityEdge, Intensity_MinIntensity, Intensity_StdIntensityEdge, Intensity_UpperQuartileIntensity, RadialDistribution_RadialCV_MaskedRed_9 of 10), while cytoplasm-related parameters (Texture_AngularSecondMoment_10_0, Texture_AngularSecondMoment_10_135, Texture_AngularSecondMoment_10_45, Texture_AngularSecondMoment_10_90, Texture_Entropy_10_135, Texture_Entropy_10_45, Texture_Entropy_10_90, Texture_SumAverage_10_0, Texture_SumAverage_10_135, Texture_SumAverage_10_45, Texture_SumAverage_10_90, Texture_SumEntropy_10_0, Texture_SumEntropy_10_135, Texture_SumEntropy_10_45, Texture_SumEntropy_10_90) contributed mainly (>0.85) to PC2. Two parameters with a high contribution to PC1 were chosen for statistical analyses (Figure 5C), and the mean radius and intensity of the mitochondria were significantly decreased by hierridin B exposure compared to DMSO.

## 3. Discussion

Cyanobacteria have a huge potential as a source of bioactive compounds relevant for the treatment of human diseases. The most famous example is brentuximab vedotin, which targets CD30 and microtubules, and is used for the treatment of malignant lymphoma after approval by the Federal Drug Agency [17]. Currently, 16 compounds derived from cyanobacteria are in different phases of clinical trials for the treatment of cancer [18]. Already-characterized modes of actions of bioactive compounds from cyanobacteria are very diverse and include classical anticancer targets, as microtubulin and actin, or non-classical targets, as histone deacetylases and proteasome [15]. Our own lab identified bioactive fractions of different marine cyanobacterial strains that demonstrated the potential to induce cytotoxicity via different molecular mode of actions on colon carcinoma cells [13]. Hierridin B was isolated from the cyanobacterium *Cyanobium* sp. LEGE06113 and showed selective cytotoxicity towards the colon adenocarcinoma cell line HT-29 [14]. In this study, the presented data indicated two possible main interactions of hierridin B with colon carcinoma cells: (1) disturbance of mitochondrial function; and (2) cell cycle arrest.

Proteomics analysis revealed a significant increase of VDAC1 protein, which was confirmed by significant increased mRNA expression of VDAC1, analyzed by real-time PCR. VDAC1 is a mitochondrial protein in the outer mitochondrial membrane at the crossroad of cellular metabolism and cell death, and is important for mitochondrial channels regulating permeability, organ volume, and apoptosis [19]. VDAC1 oligomerization forms a central pore, being regarded as a key protein for mitochondria-mediated apoptosis [20]. VDAC1 is an important target for cancer therapies and some small molecule drugs were found to target the upregulation of VDAC1, as prednisolone [21], cisplatin, [22] or arbutin [23] in different cancer cell lines. A hypothetical model suggests that an increase in Ca^2+^ enhances VDAC1 transcription, and that the resulting increase in VDAC1 protein promotes its oligomerization that in turn leads to cytochrome c release and subsequent apoptosis [19,24].

In accordance to the alterations of VDAC1, the analysis of HT-29 cells with fluorescence staining of nucleus, cytoplasm, and mitochondria demonstrated a dramatic decrease in mitochondrial staining. Since VDAC1 is involved in the formation of mitochondrial pores, it is likely that lower mitochondrial staining is related to an alteration of mitochondrial channel activities. The fluorescent probe MitoTracker CMX ROS is a live stain that is oxidized in the cells, thiol-conjugated in mitochondria and, thus, indicative of active mitochondria. Lower activity of mitochondria, as revealed by lower staining of a MitoTracker fluorescent probe, was consistent with the opening of the mitochondrial permeability transition pore (calcein AM staining) and loss of mitochondrial membrane potential (JC-1 staining), as described in photoactivated *Lonicera japonica* induced apoptosis in human lung cancer cells [25]. By using the same methodology, retinoic acid and a vitamin D analogue reduced the mitochondrial membrane potential and altered the cell cycle by increasing the apoptotic cell fraction of hepatic cancer cells [26]. In our study, the automated quantification of cytoplasm and mitochondrial parameter revealed that PCA discriminated between hierridin B treated cells and the solvent control, particularly with respect to the mitochondrial parameters as reduced mitochondrial size and fluorescence intensity, which indicated a loss of mitochondrial activity and function. However, general caution is needed with the interpretation of fluorescent probes, since some anticancer agents were described to quench the fluorescent signal without true transmembrane potential changes [27]. Still, structural motifs described to be responsible for quenching (adamantyl moieties, p-aminobenzoic acid moieties) are not found in hierridin B. Further experiments using electrophysiology of mitochondrial membrane potential would be needed to confirm the suggested loss of mitochondrial membrane potential.

Interestingly, VDAC1-interacting proteins belong to the cytoskeleton proteins (actins, tubulins), which regulate the movement of mitochondria along the cytoskeleton within the cell or the interaction of other organelles with the mitochondria. In our proteomic data, several cytoskeleton proteins had their levels decreased by the hierridin B treatment, such as GSN, TCPD, TBCA, HSPB1, and STMN1. GSN is a Ca^2+^ dependent actin-regulatory protein and inhibits VDAC1 channel activities [19]. STMN1 is involved in the mitotic spindle organization, and a knock-down of STMN1 by gene silencing increased the sensitivity towards anti-microtubule drugs and led to a cell-cycle arrest at G2/M phase [28,29]. TBCA is needed for the correct folding of β-tubulin, and gene silencing of TBCA resulted in an altered microtubule cytoskeleton, cell cycle arrest in the G1/S phase and cell death [30]. STMN1 and TBCA were strongly decreased in our study (both 10-fold), which may provide a link to the observed alterations of mRNA expression of cell cycle genes.

The reduced mRNA expression of CCNE and CCNB1 (both two-fold), and increased mRNA expression of P21CIP (10-fold) in our study indicated a general cell-cycle arrest, with known functions of CCNE for G1/S transition, of CCNB1 for G2/M transition and P21CIP for cell cycle checkpoints at G1/S and G2/M [31]. Inhibition of cell cycle progression is a common mechanism for anticancer drugs. A novel anthraquinone derivative arrested the cell cycle of HT29 colon carcinoma cells at the S phase and G2/M transition independent of P53 [32]. A low-molecular weight fucosylated chondroithin sulfate isolated from a sea cucumber induced a cell cycle arrest via increased P21CIP expression [33]. Several compounds isolated from cyanobacteria led to G2/M phase arrest (dolastatin 10, dolastatin 15) or G1/S arrest (calothrixin B, apratoxin A) as reviewed in [11]. In accordance to the data indicating cell cycle arrest, VIPR mRNA expression was decreased two-fold, which suggested a reduced cell proliferation, as described in non-small lung cancer cells [34].

Many proteomes and transcriptomes of cancer cell lines are available in publically available databases. We evaluated quantitative expression differences reported in those databases regarding the potential main targets of hierridin B effects on HT29 cells, which may be able to explain a different selectivity towards hierridin B exposure. The Human Protein Atlas (http://www.proteinatlas.org/) and NCI-60 proteome database (http://129.187.44.58:7070/NCI60/) do not provide relevant quantitative differences in various cancer cell lines for these targets. From the Cancer Cell Encyclopedia (http://portals.broadinstitute.org/ccle/home), a dataset (GEO Accession: GSE36133) is available containing comparative microarrray data across cancer cell lines, but expression differences do not justify a different selectivity. In the Colorectal Cancer Atlas (http://www.colonatlas.org/), sequence variants are described for VDAC1 and STMN1 in HT29 and RKO cells. A sequence variance for VDAC1 in HT29 cells is at a splice site (p.G23_splice), while a sequence variance for STMN1 in RKO cells is a stop gain (c.28A > C). Further studies will be needed to explain the selectivity of hierridin B in HT29 cells.

In summary, the exposure of HT-29 carcinoma cells to hierridin B induced significant changes in VDAC1 mRNA expression and protein content. Fluorescence microscopy and automated quantification of mitochondria by CellProfiler demonstrated that mitochondrial activity was decreased. The lower protein level of interacting cytoskeleton proteins (STMN1, TBCA) provided a link to the altered cell cycle genes (CCNE, CCNB1, P21CIP) and lower cell proliferation (VIPR). Therefore, hierridin B is a marine cyanobacterial metabolite that likely targets mitochondrial function. 

## 4. Materials and Methods

### 4.1. Cyanobacteria Culture and Isolation of Hierridin B

The cyanobacterial strain *Cyanobium* sp. LEGE06113 was isolated from the Portuguese coast and is maintained in the Blue Biotechnology and Ecotoxicology Culture Collection [35] at the Interdisciplinary Centre of Marine and Environmental Research (CIIMAR/CIMAR), Porto, Portugal. The culture conditions of this cyanobacterium, subsequent isolation and purification of hierridin B were described in [14], and yielded 240 μg of isolated hierridin B available for the presented work.

### 4.2. HT-29 Assays

The colon adenocarcinoma cell line HT-29 is from human origin and was obtained from the American Type Culture Collection (ATCC) and cultured as described in [12]. Briefly cells were cultured in DMEM Glutamax medium (Dulbecco’s Modified Eagle Medium DMEM GlutaMAX™), supplemented with 10% (*v*/*v*) fetal bovine serum, 1% of penicillin-streptomycin (Pen-Strep 100 IU/mL and 10 mg/mL, respectively) and 0.1% fungicide Amphotericin B (250 μg/mL). Cells were maintained in a humidified incubator at 37 °C, with 5% CO_2_. The IC_50_ value of hierridin B on the HT-29 cell line was 100.2 μM or 36.4 μg/mL [14], and this concentration was used for mRNA expression, proteomics and fluorescent microscopy analysis. Cells were seeded in 24-well plates at a concentration of 1.3 × 10^5^ cells/cm^2^ and exposed for 48 h to hierridin B. For mRNA expression assays, proteomics and fluorescent microscopy six, four, and three replicates were prepared, respectively.

### 4.3. Protein Extraction and Two-Dimensional Gel Electrophoresis (2DGE)

Protein extraction from the cell pellet was performed by adding 80 μL solubilisation buffer (urea (7 M), thiourea (2 M), 3-[(3-Cholamidopropyl)dimethylammonio]-1-propanesulfonate hydrate (CHAPS, 4%, *w*/*v*), dithiothreitol (65 mM), and ampholytes (0.8%, *v*/*v*, pH 4–7)) on the cell pellets. After one hour homogenization, solutions were centrifuged at 16,000× *g* for 20 min at 4 °C. The supernatant was collected, and total protein content determined by the Bradford method [13]. 2DGE was performed for protein separation following the procedure described in [13]. Two-hundred twenty-five micrograms of protein in a volume of 250 μL solubilizaton buffer were loaded on 11 cm, pH 3–10 gel strips and separated by isoelectric focusing (IEF) using Protean IEF Cell (Bio-Rad) and 12% (*w*/*v*) acrylamide SDS-PAGE with SE900 Hoefer.

### 4.4. Gel Staining, Image Acquisition, and Protein Expression Analysis

Gel staining was performed using the Coomassie Blue Colloidal method, gel images were acquired in the GS-800 calibrated densitometer from Bio-Rad, and protein spots were detected automatically with the PDQuest 2-D analysis software, according to [13]. The quantitative variations were statistically evaluated by Student’s *t* and Mann-Whitney U tests (*p* ≤ 0.05).

### 4.5. Protein Identification

Protein identification was performed according to the method described by [36]. Briefly, protein spots were excised from gels, reduced with dithiothreitol (DTT, Sigma, St. Louis, MO, USA), alkylated with iodacetamide (Sigma) and subjected to in-gel digestion using trypsin (Promega, Madison, WI, USA). The resulting tryptic peptides were desalted and concentrated using reversed-phase C18 10 μL tips (Pierce, Thermo Fisher Scientific, Waltham, MA, USA), according to the manufacturer’s instructions. The peptides were eluted directly onto the MALDI plate in duplicates using the matrix alpha-cyano-4-hydroxycinnamic acid (7–8 mg/mL) prepared in acetonitrile (50%, *v*/*v*) and trifluoroacetic acid (0.1%, *v*/*v*). Peptide mass spectra were obtained on a MALDI-TOF/TOF mass spectrometer (4800 Proteomics Analyzer, Applied Biosystems/Life Technologies, Carlsbad, CA, USA) in the positive ion reflector mode. Spectra were obtained in the mass range between 800 and 4500 Da with ca. 1500 laser shots. For each sample spot, a data dependent acquisition method was created to select the six most intense peaks, excluding those from the matrix, trypsin autolysis, or acrylamide peaks, for subsequent MS/MS data acquisition. Spectra were processed and analyzed by the Global Protein Server Workstation (Applied Biosystems), which uses internal MASCOT software (v2.1.0 Matrix Science, London, UK) on searching the peptide mass fingerprints and MS/MS data. The Swiss-Prot non-redundant protein sequence database (October 2014) was used for all searches under taxonomy *Homo sapiens*. Database search parameters are as follows: carbamidomethylation and propionamide of cysteine as a variable modification, as well as oxidation of methionine, and the allowance for up to two missed tryptic cleavages. The peptide mass tolerance was 25 ppm and the fragment ion mass tolerance was 0.3 Da. Positive identifications were accepted up to a 95% confidence level. Spots with no identification were repeated in duplicates using the same conditions. The free online software STRING [37] was used for evaluation of protein interaction networks between the solvent control and the hierridin B exposure groups. Protein interaction analysis regarding their biological processes were performed with ClueGo + CluePedia [38].

### 4.6. Real-Time Analysis

For real-time PCR, two reference genes (RPL8, HPRT1) and four target genes (CCNB1, CCNE, P21CIP, BAD) for apoptosis and cell cycle were selected, as described in [13]. Furthermore, real-time PCR assays were developed for target genes involved in mitochondrial regulation (VDAC), tumor survival (SHMT2), as apoptosis (TRAIL), and cell proliferation (VIPR) (Table 2). RNA was extracted using the Nucleospin RNA XS Kit (Macherey-Nagel, Dueren, Germany), quantified with the Qubit Fluorometer (Invitrogen, Carlsbad, CA, USA) according to the manufacturer instruction and RNA quality was assessed by 1% gel agarose electrophoresis. Transcription of RNA into cDNA was performed by reverse transcription (RT) using the iScript Reverse Transcription Supermix Kit (BioRad). One microgram of total RNA was reverse-transcribed in a 20 μL reaction (5 min at 25 °C, 30 min at 42 °C and 5 min at 85 °C), and cDNA was stored at −20 °C.

Real-time PCR analyses were performed using the iQ5 real-time PCR machine (Bio-Rad). The final reaction volume was 20 μL containing 1xiQ SYBR Green Supermix (Bio-Rad), each 200 nM forward and reverse primer, and 2 μL cDNA sample (1:10 diluted), and samples were run as described in [13].

### 4.7. Fluorescence Microscopy

Cells were seeded on glass slides inserted into a 24-well plate at a concentration of 1.3 × 10^5^ cells/cm^2^ exposed for 48 h to hierridin B or solvent control (DMSO) as described above. Three replicate wells were used for the control group (DMSO) and two replicate wells for hierridin B, due to limitations in compound availability. Cells were stained for 20 min at 37 °C with 5 μg/mL Hoechst 33342 (HO, nucleus; Sigma) and 250 nM MitoTracker RedCMXRos (mitochondria; Life Technologies) and then fixed in 4% paraformaldehyde. Following, cells were permeabilized with 0.5% Triton X-100 in PBS and stained with 100 nM Actin-stain 488 phalloidin (actin fibers, Cytoskeleton Inc., Denver, CO, USA) for 30 min at room temperature in the dark. The glass slides were then mounted on microscope slides with Fluoromount (Sigma), before being visualized on a fluorescence microscope (Olympus BX-71). Eight to 13 images were taken randomly per replicate at a magnification of 400× with fixed exposure times (HO: 200 ms, MitoTracker: 714 ms, Actin: 50 ms), which summed up to 29 images for DMSO group, and 24 images for the hierridin B group. Images were analyzed with the CellProfiller software [39] and, in total, 160 parameters related to cytoplasm and mitochondria were automatically quantified. The following modules were included in the pipeline: object intensity (19 parameters), object radial distribution (three parameters, in 10 bins each), size and shape of objects (18 parameters), and texture (13 parameters). A complete list of parameters can be found in the manual of CellProfiler (available at [40]). Values of parameters from individual cells on each image were averaged, and the mean value per image of the corresponding parameter was used for further statistical analyses.

### 4.8. Statistics

For the real-time PCR data, the Kolmogorov-Simirnov test was used to analyze the normality distribution, and the Bartlett’s test for equal variances. If these conditions were met, one-way ANOVA was performed followed by Tukey’s post-hoc test. If conditions for parametric tests were not fulfilled, non-parametric tests, Kruskal-Wallis, and Dunn’s post-hoc test were used. Differences were considered significant when *p* ≤ 0.05. For the fluorescence microscopy data quantified with CellProfiller, principal component analysis was performed for the 160 parameters of cytoplasm and mitochondria. Factors were Kaiser normalized and Varimax rotated. Variables that contributed >0.85 to the factor loadings of PC1 or PC2 were considered as important.

## Figures and Tables

**Table 1:** Significant regulated proteins of HT-29 cells exposed to hierridin B compared with the control group (DMSO).

SPP	Protein Name	DMSO (Mean ± SD)	Hierridin B (Mean ± SD)	Fold (×)	Acession Number	Gene	Protein Score	Matched Peptides MS MS/MS	
506	Calreticulin	3316 ± 504	1951 ± 172	0.6	CALR_HUMAN	CALR	215	15	2
1513	Diablo homolog, mitochondria	717 ± 23	502 ± 12	0.7	DBLOH_HUMAN	DIABLO	105	7	2
2602	Tumor protein D52	946 ± 96	633 ± 146	0.7	TPD52_HUMAN	TPD52	133	8	2
2713	-	281 ± 80	76 ± 70	0.3	-	-	-	-	-
3101	Tubulin-specific chaperone A	730 ± 313	88 ± 18	0.1	TBCA_HUMAN	TBCA	130	6	3
3113	-	1504 ± 226	148 ± 142	0.1	-	-	-	-	-
3304	Stathmin	700 ± 179	95 ± 67	0.1	STMN1_HUMAN	STMN1	85	5	1
3507	-	2247 ± 768	6225 ± 2081	2.8	-	-	-	-	-
3514	UMP-CMP kinase	721 ± 148	240 ± 243	0.3	KCY_HUMAN	CMPK1	100	5	2
4704	Serum albumin	6705 ± 611	3282 ± 1123	0.5	ALBU_HUMAN	ALB	74	4	1
4802	Gelsolin	743 ± 239	355 ± 192	0.5	GELS_HUMAN	GSN	178	17	2
4901	Neutral alpha-glucosidase AB	577 ± 178	153 ± 69	0.3	GANAB_HUMAN	GANAB	304	23	3
5601	Heat-shock protein beta-1	680 ± 70	517 ± 75	0.8	HSPB1_HUMAN	HSPB1	128	3	1
5801	Delta(3,5)-delta(2,4)-dienoyl-CoA isomerase, mitochondrial	980 ± 40	1368 ± 230	1.4	ECH1_HUMAN	ECH1	200	10	3
6105	Ubiquitin-like protein ISG15	2519 ± 237	884 ± 473	0.4	ISG15_HUMAN	ISG15	80	4	1
6202	Alpha-enolase	9861 ± 410	7341 ± 1801	0.7	ENOA_HUMAN	ENO1	499	23	3
7112	-	1293 ± 155	232 ± 131	0.2	-	-	-	-	-
7305	Serine hydroxymethyl transferase, mitochondrial	718 ± 113	308 ± 93	0.4	GLYM_HUMAN	SHMT2	277	17	2
7803	Elongation factor 2	511 ± 90	181 ± 92	0.4	EF2_HUMAN	EEF2	304	22	3
7804	Elongation factor 2	811 ± 236	405 ± 128	0.5	EF2_HUMAN	EEF2	307	26	3
8404	t-complex protein 1 subunit delta	543 ± 163	272 ± 112	0.5	TCPD_HUMAN	CCT4	158	12	3
8802	Voltage-dependent anion-selective channel protein 1	2319 ± 695	3487 ± 162	1.5	VDAC1_HUMAN	VDAC1	111	8	1

**Table 2:** Primers selected for mRNA expression as target genes.

Gene	Name	Primer Sequence	Efficiency	*R*^2^	Anneling Temperature
VDAC	Voltage-dependent anion channel 1	F: CGGAAGGCAGAAGATGGC	101.6%	0.936	57 °C
		R: TTGGTGGTCTCAGTGTTGG			
SHMT2	Serine Hydroxymethyl transferase 2	F: CTGCGACTTCCGAGTTGCGATG	101.6%	0.963	57 °C
		R: GGCTGCGTTGCTGTGCTGAG			
TRAIL	Tumor Necrosis factor superfamily, member 10	F: TCTCTCTGTGTGGCTGTAAC	97.9%	0.992	57 °C
		R: TCATACTCTCTTCGTCATTGGG			
VIPR	Vasoactive Intestinal Peptide receptor 1	F: CACCATCAACTCCTCACTG	105.7%	0.992	57 °C
		R: CTGCTGTCACTCTTCCTG			
